# Cumulative evidence for association of rhinitis and depression

**DOI:** 10.1186/s13223-021-00615-5

**Published:** 2021-10-24

**Authors:** Jing Wang, Dongqiong Xiao, Huayou Chen, Juan Hu

**Affiliations:** 1 Department of Radiology, The First Hospital of Jilin University, Changchun, 130021 Jilin China; 2 Department of Emergency, Key Laboratory of Birth Defects and Related Diseases of Women and Children (Sichuan University), West China Second University Hospital, Sichuan University, Ministry of Education, Chengdu, 610041 China; 3 Department of Paediatrics, The First People’s Hospital of Zigong, Zigong, 643000 China

**Keywords:** Rhinitis, Depression, Allergic rhinitis, Asthma

## Abstract

**Background:**

Several primary studies evaluated the association between rhinitis and the incidence of depression and yielded inconsistent results. We conducted a meta-analysis of studies evaluating the association between rhinitis and depression.

**Methods:**

We searched the EMBASE, PubMed and Cochrane Library databases for studies published in English before April 1, 2019. The studies were included if they reported any type of rhinitis in relation to depression. Two authors independently extracted the data. The odds ratios (ORs) were pooled using a random-effects model. Stratified analyses were conducted to evaluate the association.

**Results:**

Among the 3472 initially identified studies, we included 14 studies involving a total of 19.36 ± 1.1 million participants according to predefined inclusion criteria. The associations between rhinitis (R), allergic rhinitis (AR), and nonallergic rhinitis (NAR) and depression were significant with ORs of 1.86 (95% CI 1.32 to 2.62, *p* < 0.05), 1.54 (95% CI 1.24 to 1.90, *p* < 0.05), and 2.15 (95% CI 1.49 to 3.09, *p* < 0.05), respectively. The results were consistent and statistically significant in all subgroup analyses.

**Conclusions:**

Rhinitis was associated with an increased risk of depression. Further prospective studies involving large sample sizes are required to confirm the results by considering more confounders and clarify the mechanisms.

**Supplementary Information:**

The online version contains supplementary material available at 10.1186/s13223-021-00615-5.

## Background

Rhinitis, including allergic rhinitis (AR) and nonallergic rhinitis (NAR), is highly prevalent globally [1–3]. The prevalence of AR ranges from 10 to 40% worldwide [4–6], and approximately 60 million people are affected by AR and 20 million people are affected by NAR in the United States [7–9]. The magnitude of this public health challenge is increasing, and estimates suggest that at least 1 in 4 people may suffer from rhinitis. The treatments for different forms of rhinitis may differ according to the pathogenesis. The severity and persistence of rhinitis may impact the quality of life and, thus, result in many cognitive and emotional adverse events, anxiety and depression [1]. In theory, effective treatments for rhinitis may reduce the risk of developing emotional adverse events.

The prevalence of depression is also increasing, and access to effective treatments remains limited [10], representing a concerning trend given that depression imposes a significant public health burden and large demand on health care systems [11]. Depression treatments are categorized into the following methods: psychotherapy with or without antidepressants and antidepressants only [12].

Several primary studies [1, 3, 5–7, 13–18] evaluated the association between rhinitis and the incidence of depression and yielded inconsistent results. Lu et al. [19] reviewed allergic disorders and the risk of depression and allergic disorders, including AR. Additionally, Sansone et al. [20] published a systematic review investigating the relationships between AR and mood syndromes, and 10 of 12 studies showed a positive relationship. Although previous systematic reviews/meta-analyses studied the association between AR and depression, this manuscript is original because this meta-analysis investigates the association between NAR and depression. Therefore, we conducted a meta-analysis to evaluate the association among rhinitis, AR, NAR and depression.

## Methods

### Retrieval of studies

The reporting of this meta-analysis of observational studies is consistent with the Meta-Analysis of Observational Studies in Epidemiology (MOOSE) guidelines (Additional file 1). The PubMed, EMBASE, and Cochrane Library databases were searched through April 1, 2019. The search consisted of the following two terms: rhinitis and depression. We used the following key words to search for the first term: “rhinitis” OR “rhinit*” OR “NARES” OR “NAR” OR “LAR” OR “NANIPER”. We used the following key words to search for the second term: “depression” OR “depressive disorder” OR “depressive disorder, major” OR “dysthymic disorder” OR “depress*” OR “melancholia”. In addition, we used “AND” to connect the two terms (for the search strategy, see 10.17632/ccvm3cvbtm.2). The retrieved studies were first screened by reading the titles and abstracts. Two authors (Jing Wang and Dongqiong Xiao) subsequently read the full texts of the remaining publications independently. A third author (Huayou Chen) resolved any disagreements (Additional file 3).

### Definitions

AR is an IgE-mediated inflammatory disease of the nasal mucosa characterized by the presence of one or more nasal symptoms, including itching, sneezing, nasal discharge and nasal blockage [21, 22]. NAR is not IgE-mediated rhinitis and consists of at least the following 2 subtypes: a non-eosinophilic subtype and an eosinophilic subtype [23]. Rhinitis is a general term referring to various nose AR/NAR inflammation and infections (viral sinusitis). Depression is based on a highly variable set of symptoms rather than objective diagnostic tests. A diagnosis of major depression was made when a certain number of symptoms listed in the Diagnostic and Statistical Manual (DSM-5) were reported for longer than 2 weeks [24].

### Study selection

The inclusion criteria were as follows: (1) studies involving participants investigated for any of the following outcomes: the incidence, prevalence, risk or odds ratio (OR) of depression among rhinitis, AR, NAR and control participants; (2) studies evaluating the association between rhinitis and depression and reporting the unadjusted and/or adjusted ORs and their corresponding 95% confidence intervals (CIs), unadjusted and/or adjusted OR estimates and 95% CIs, or the number of exposed and unexposed participants; and (3) studies published in English with a case–control, cohort, or cross-sectional design.

The exclusion criteria were as follows: (1) studies reporting the results of only animal experiments; (2) unrelated studies or studies in which the data overlapped with those of another study; or (3) reviews, case reports, meta-analyses and letters.

### Data extraction

The data were independently extracted from the studies by two reviewers (Jing Wang and Dongqiong Xiao) and aggregated using a standardized form; the collected data included the study author, publication year, study design, study location, sample size, age, ascertainment of rhinitis, type of rhinitis, ascertainment of depression, depression diagnostic criteria, primary outcomes, confounding factors, data source, and Newcastle–Ottawa Scale (NOS) score.

### Quality evaluation

The methodological quality of all included studies (Additional file 2: Table S1) was examined using the NOS [25] by two reviewers (Jing Wang and Dongqiong Xiao) independently, and a third author resolved any disagreements. The reviewers assessed the quality scores (varying from 0 to 9) in the following three domains: selection of the study population, comparability, and evaluation of exposure and outcomes.

### Statistical analysis

The ORs and 95% CIs were used as measures of the association between rhinitis and depression across the studies. In the case of original studies comparing the number of participants who developed depression following exposure to rhinitis compared with control groups, we calculated the ORs and 95% CIs of each study. All data from the included studies were converted into log(ORs) and standard errors (SEs) [26]. We pooled the log(ORs) and SEs of each study separately using the DerSimonian–Laird formula (random effects model) [27]. The statistical heterogeneity [28] among the studies was assessed using the *I*^2^ statistic [29]. Values of *I*^2^ > 50% and *p* < 0.1 indicated high heterogeneity [30].

We conducted stratified analyses based on the study location (Asia, the United States, Europe, or other countries), study design (cohort or cross-sectional), sample size (≥ 10,000 or ˂10,000), sample population (< 18 years or ≥ 18 years), ascertainment of depression (self-reported or diagnosed), ascertainment of rhinitis (self-reported or diagnosed), study quality (NOS score > 5 or NOS score ≤ 5), adjustment for confounding factors (≥ 8 factors or ≤ 7 factors), adjustment for age (yes or no), adjustment for sex (yes or no), adjustment for asthma (yes or no), adjustment for atopic dermatitis (yes or no), adjustment for household income (yes or no), and adjustment for comorbidities (yes or no).

We used funnel plots [31], Egger’s tests and Begg’s tests in the meta-analysis to assess publication bias. We used Stata software version 12.0 (StataCorp, College Station, TX) to perform the statistical analyses.

## Results

### Literature search

**Fig. 1:**
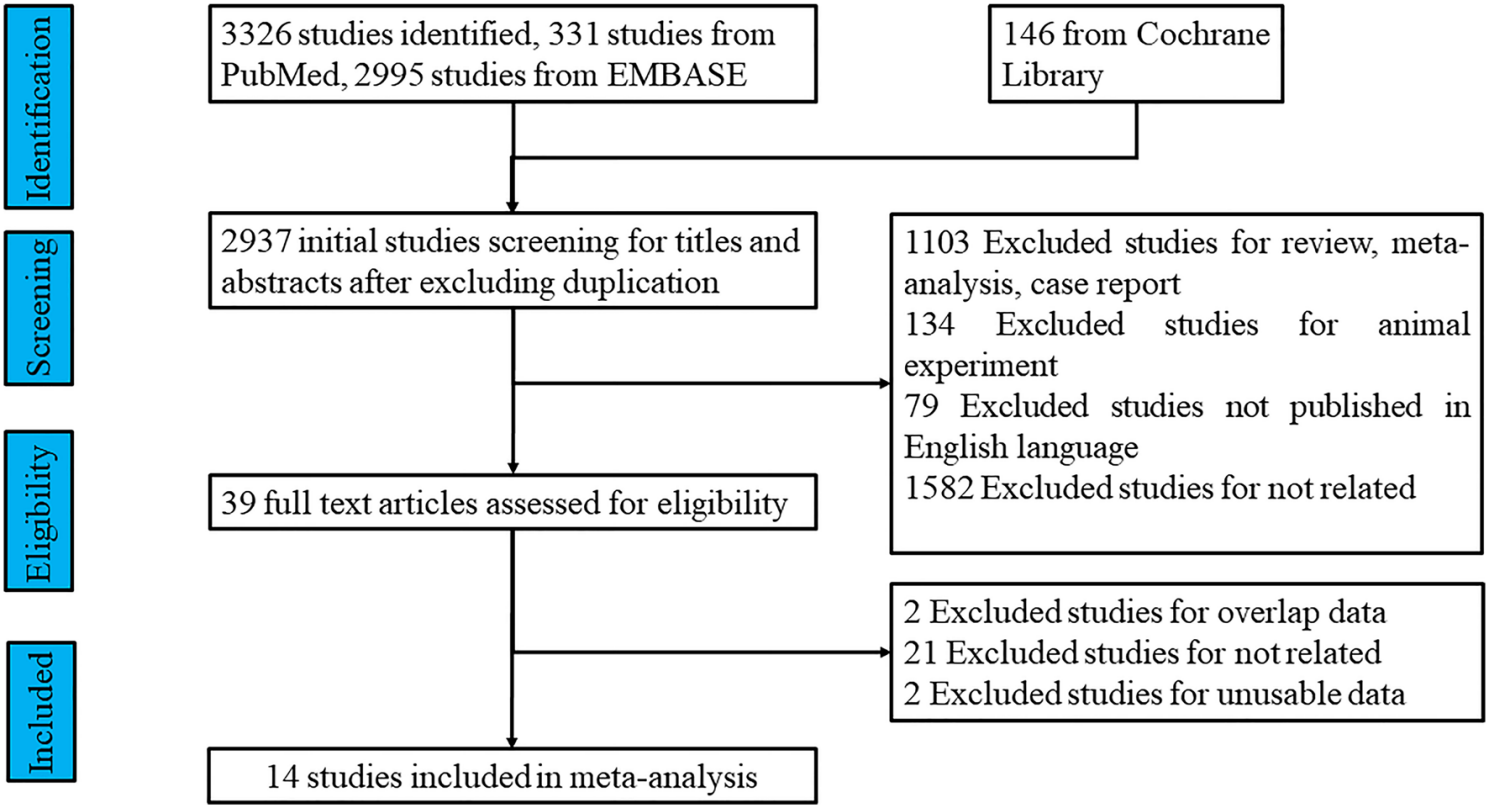
Flow chart describing the study selection

We identified 3472 potential studies, including 331 studies from PubMed, 146 studies from the Cochrane Library, and 2995 studies from EMBASE (10.17632/ccvm3cvbtm.2). After careful screening, 3458 studies were excluded for the reasons listed in Fig. 1, and 14 studies reporting the association between rhinitis and depression met the final inclusion criteria (see Fig. 1). The characteristics of these 14 included studies (study author, publication year, study location, study design, sample size, participants, ascertainment of rhinitis, type of rhinitis, diagnosis of rhinitis, ascertainment of depression, depression diagnostic criteria, outcomes, confounding factors, and data source) are summarized in Table 1.

**Table 1:** Characteristics of the included studies

Study	Publication year	Study location	Study design	Sample size	Participants	Ascertainment of rhinitis	Type of rhinitis	Diagnosis of rhinitis	Ascertainment of depression	Depression diagnostic criteria	Outcomes	Confounding factors	Data source
Zhou	2017	United States	Cross-sectional	19.1 ± 1.1 million	Adults	Survey participants self-reported	AR	NA	Survey self-reported	NA	1.32 (1.12–1.54)	Region, sex, race, income, education, and age	2014 (NHIS)
Yamamoto-Hanada	2019	Japan	Cross-sectional	86,085	Pregnant women	Self-reported	Rhinitis	NA	Self-reported	K-6 ≥ 5	1.2 (1.17–1.24)	NA	NA
Roxbury	2019	United States	Cross-sectional	4320	Adults	Self-reported	Rhinitis/AR/NAR	NA	Self-reported	PHQ-9 ≥ 10	1.42 (1.03–1.95)/0.9 (0.68–1.18)/1.99(1.34–2.96)	Age, gender, race, obesity comorbid medical conditions, including self-reported history of asthma, coronary artery disease, heart attack, stroke, thyroid disease, emphysema, chronic bronchitis, and trouble seeing; and self-rated general health condition (excellent, very good, good, fair, or poor)	2005–2006 (NHANES)
Kim	2016	Korea	Cross-sectional study	11,154	> 19 y	Self-reported	AR	NA	Self-reported	EuroQol 5-dimension (EQ-5D)	2.585 (1.519–4.398)/severe 1.49 (0.894–2.481) 1.254 (0.897–1.752)	Age, sex, BMI, smoking status, alcohol use status, exercise status, region of residence, income, education, marital status, asthma, and atopic dermatitis	KNHANES, 2011–2012
Nanda	2016	United States	Prospective cohort	546	4–7 y	Diagnosed	AR	1 aeroallergen SPT positive+	Diagnosed	BASC-2	3.2 (1.7–6.5)	Gender, parental asthma, maternal education, BMI, and sleep disturbance	2001–2003, CCAAPS
Chen	2013	Taiwan	Prospective cohort study	1673	12-15y	Diagnosed	AR	ICD-9-CM	Diagnosed	ICD-9-CM	1.59 (1.02–2.50)	Residence location and comorbid allergic diseases	1996–2000, NHIRD
Bedolla-Barajas	2017	Mexico	Cross-sectional study	241	≥ 18 and ≤ 50	Diagnosed	AR/NAR		Diagnosed	BDI-II > 13	2.5 (1.24–4.9)/3.3 (1.28–8.25)	Sex	2012–2013
Valero	2015	Spain	Cross-sectional study	670	> 18 y	Self-reported	Rhinitis	NA	Self-reported	HAD	17.6% vs. 4.76%	NA	NA
Wei	2016	Taiwan	Cohort	5075/44729	10–17 y	Diagnosed	Rhinitis	ICD-9-CM	Diagnosed	ICD-9-CM	1.8 [1.29–2.50]	Demographic data	1995–2000, NHIRD
Audino	2014	Italy	Cross-sectional study	1283	10–13 y	Self-reported	Rhinitis	NA	Diagnosed	TAD > 115	17.7% vs. 11.2%	NA	Palermo, Italy
Derebery	2008	United States	Cohort	7024	> 18 y	Self-reported	Rhinitis	NA	Self-reported	NA	17.2% (3831) vs. 8.3% (3193)	NA	2004 NFO
Tas	2019	Turkey	Cross-sectional	101/74	> 18 y	Diagnosed	AR	Endoscopic nasal examination	Self-reported	TEMPS-A	23.8% (24/101) vs. 10.8% (8/74)	NA	2017–2018
Shin	2018	Korea	Cross-sectional study	15,441	> 19 y	Self-reported	AR	NA	Self-reported	HRQoL	1.72 (1.08–2.73)	Age, gender, educational level, marital status, household income, occupation, residential area, smoking status, alcohol consumption, exercise status, sleep duration, and obesity; comorbidities, such as hypertension, diabetes, stroke, ischemic heart disease, arthritis, asthma, atopic dermatitis, thyroid disease, liver cirrhosis, chronic renal failure, and depression; activity limitations; subjective health; and total cholesterol and triglyceride levels	2013–2015 KNHANES
Seo	2012	Korea	Cross-sectional	76,937	Adolescents	Self-reported	AR	NA	Self-reported	NA	1.14 (1.08–1.20)	NA	2009 Korean Youth’s Risk Behavior Web-based Study

*AR* allergic rhinitis, *BASC-2* Behavior Assessment System for Children, Second Edition, *BDI-II* Beck Depression Inventory-II, *CCAAPS* Cincinnati Childhood Allergy and Air Pollution Study, *CI* confidence interval, *EPOS* European Position Paper on Rhinosinusitis and Nasal Polyps, *HADS* Hospital Anxiety and Depression score, *ICD-9-CM* International Classification of Disease, 9th Revision, Clinical Modification, *K-6* Kessler’s K-6 Non-Specific Psychological Distress Scale, *KNHIS-NSC* National Sample Cohort of the Korea National Health Insurance Service, *TAD* testing for depression and anxiety characteristics using the Depression and Anxiety in Youth Scale, *KNHANES* Korean National Health and Nutrition Examination Survey, *NA* not applicable because only 1 study, *NAR* nonallergic rhinitis, *NFO* National Family Opinion, *NHANES* National Health and Nutrition Examination Survey, *NHIRD* National Health Insurance Research Database, *NHIS* National Health Interview Survey, *NHIRD* National Health Insurance Research Database, *NPHS* National Population Health Survey, *OR* odds ratio, *QOL* quality of life, *TEMPS-A* Temperament Evaluation of Memphis, Pisa, Paris, France, San Diego Auto-questionnaire

### Characteristics and quality of the included studies

The characteristics of the fourteen included studies are shown in Table 1. Among the included studies, four studies [1, 17, 32, 33] were cohort studies, and ten studies [5–7, 13, 14, 34–38] were cross-sectional studies. The association between rhinitis and depression was the primary outcome of interest in six studies [7, 32–34, 37, 38], the association between AR and depression was the primary outcome in nine studies [1, 5–7, 13, 17, 35, 36, 39], and the association between NAR and depression was the primary outcome in two studies [7, 35].

The included studies were published between 2008 and 2019, and the number of participants ranged from 175 to 19.1 ± 1.1 million, yielding a total of 19.36 ± 1.1 million participants across the studies.

Four studies [7, 13, 17, 33] were conducted in the United States, six studies [1, 6, 14, 32, 34, 36] were conducted in Asia, three studies [5, 37, 38] were conducted in Europe, and one study [35] was conducted in Mexico. Five studies [1, 17, 32, 36, 38] involved children or adolescents, one study [34] involved only pregnant women, and the other eight studies [5–7, 13, 14, 33, 35, 37] included both adult men and women.

Among the included studies, four studies [6, 7, 13, 14] adjusted for age, two studies [14, 17] adjusted for asthma, and three studies adjusted for more than 8 confounding factors [6, 7, 14].

The quality scores of the included studies ranged from 5 to 8 (10.17632/ccvm3cvbtm.2), and the quality scores were considered high.

### Quantitative results (meta-analysis)

**Fig. 2:**
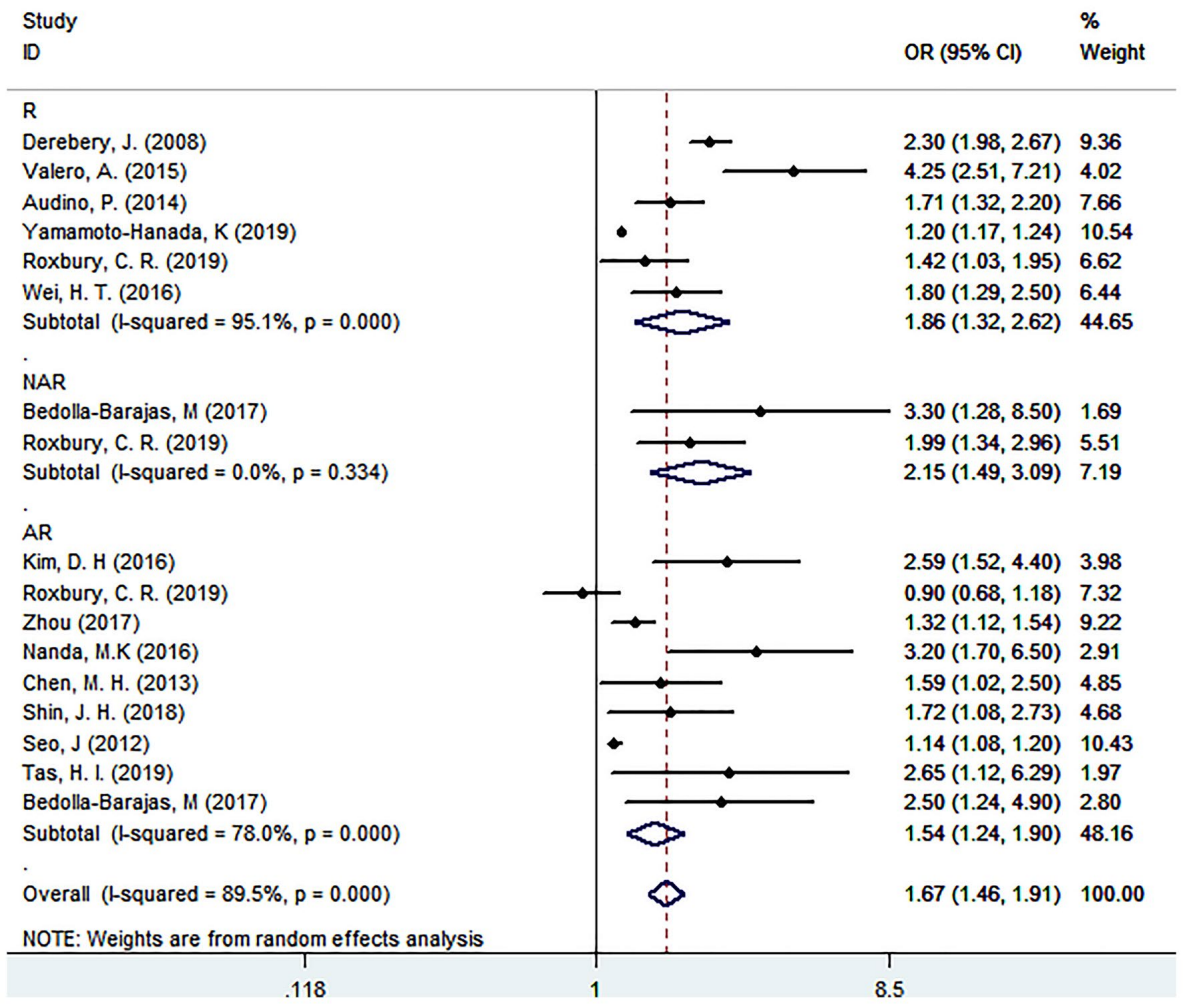
Forest plot of the pooled odds ratio of the association between any type of rhinitis and depression

Among the 14 selected studies, one study [7] found a nonsignificant association between AR and depression, while the other studies revealed an association between rhinitis, AR, or NAR and a significantly increased risk of depression. Two studies [34, 36] reported unadjusted ORs, eight studies reported adjusted ORs, and four studies [5, 33, 37, 38] reported the number of participants with and without rhinitis who developed depression. All 14 studies reported the risks as ORs, ranging from 0.9 to 4.25. Any type of rhinitis was associated with an increased risk of depression compared with the control with a pooled OR of 1.67 (95% CI 1.46, 1.91). High heterogeneity was found in these studies (I^2^ = 89.5%, *p* < 0.001) (Fig. 2).

Two included studies [7, 35] (number of participants = 4561) reported a significant association between NAR and depression with a pooled OR of 2.15 (95% CI 1.49, 3.09). No significant heterogeneity was found in these studies (I^2^ = 0, *p* = 0.334) (Fig. 2).

### Stratified analyses

#### Allergic rhinitis and the risk of depression

Among the fourteen studies included in our analysis, nine articles reported an association between AR and depression. Stratified analyses of several key study characteristics and clinical factors were performed to evaluate the possible sources of heterogeneity in the included studies (Table 2). The association between AR and depression was significant (OR: 1.54, 95% CI 1.24–1.90), and this association was consistent in all stratified analyses (Table 2). Stronger associations between AR and depression were found in the cohort studies (OR: 2.15, 95% CI 1.09–4.25) compared with the cross-sectional studies (OR: 1.42, 95% CI 1.14–1.75), in the studies with small sample sizes (< 10,000) (OR: 1.86, 95% CI 1.08–3.18) compared to the studies with large sample sizes (≥ 10,000) (OR: 1.41, 95% CI 1.12–1.78), and in the studies conducted in Europe and other countries compared with the studies conducted in the United States and Asia (Table 2).

**Table 2:** Stratified analysis of the associations between rhinitis and depression

Variables	Allergic rhinitis and depression			*p*	Rhinitis and depression			*p*
	Studies	OR (95% CI)	*I*^2^ (*p*-value)		Studies	OR (95% CI)	*I*^2^ (*p*-value)	
Total	9	1.54 (1.24, 1.90)	78 (< 0.05)		6	1.86 (1.32, 2.62)	95.1 (< 0.05)	
Study location								
Europe	1	2.65 (1.12, 6.28)	NA	**< 0.05**	2	2.61 (1.07, 6.37)	89.2 (< 0.05)	**< 0.05**
Unites States	3	1.40 (0.88, 2.21)	85.1 (< 0.05)		2	1.85 (1.15, 2.96)	86.1 (< 0.05)	
Asia	4	1.60 (1.09, 2.33)	78.3 (< 0.05)		2	1.42 (0.96, 2.10)	82.5 (< 0.05)	
Other countries	1	2.5 (1.26, 4.97)	NA		0	NA	NA	
Study design								
Cohort	2	2.15 (1.09, 4.25)	65.4 (> 0.05)	**< 0.05**	2	2.13 (1.71, 2.66)	42.9 (> 0.05)	> 0.05
Cross-sectional	7	1.42 (1.14, 1.75)	76.9 (< 0.05)		4	1.75 (1.19, 2.55)	90 (< 0.05)	
Sample size								
≥ 10,000	4	1.41 (1.12, 1.78)	79.1 (< 0.05)	**< 0.05**	2	1.42 (0.96, 2.10)	82.5 (< 0.05)	**< 0.05**
< 10,000	5	1.86 (1.08, 3.18)	80.6 (< 0.05)		4	2.10 (1.51, 2.90)	81.9 (< 0.05)	
Sample population								
Adult	6	1.63 (1.17, 2.26)	75.7 (< 0.05)	**> **0.05	4	1.93 (1.21, 3.10)	96.7 (< 0.05)	> 0.05
< 18 years	3	1.64 (0.98, 2.77)	81.9 (< 0.05)		2	1.74 (1.42, 2.13)	0 (0.81)	
Ascertainment of depression								
Self-report	6	1.34 (1.09, 1.65)	76.4 (< 0.05)	**< 0.05**	4	1.93 (1.21, 3.10)	96.7 (< 0.05)	**> **0.05
Diagnosed	3	2.19 (1.42, 3.38)	38.3 (> 0.05)		2	1.74 (1.42, 2.13)	0 (0.81)	
Ascertainment of rhinitis								
Self-report	5	1.29 (1.05, 1.57)	77.4% (< 0.05)	**< 0.05**	5	1.88 (1.27, 2.77)	95.9 (< 0.05)	**> **0.05
Diagnosed	4	2.20 (1.56, 3.08)	14.3% (> 0.05)		1	1.8 (1.29, 2.51)	NA	
Study quality								
NOS score > 5	6	1.6 (1.17, 2.18)	77.3% (< 0.05)	**> **0.05	3	1.39 (1.09, 1.78)	70.4 (< 0.05)	**< 0.05**
NOS score ≤ 5	3	1.79 (0.93, 1.90)	76.8% (< 0.05)		3	2.37 (1.66, 3.39)	80.1 (< 0.05)	
Adjusted for confounding factors								
Minimal (≤ 7 factors)	6	1.56 (1.22, 1.99)	79.8 (< 0.05)	**> **0.05	5	1.97 (1.32, 2.93)	96.1 (< 0.05)	**< 0.05**
Substantial (≥ 8 factors)	3	1.54 (0.81, 2.94)	86.2 (< 0.05)		1	1.42 (1.03, 1.95)	NA	
Adjusted for age								
Yes	4	1.43 (1.0, 2.03)	79.6% (< 0.05)	**< 0.05**	1	1.42 (1.03, 1.95)	NA	**< 0.05**
No	5	1.9 (1.2, 3.01)	79.4% (< 0.05)		5	1.97 (1.32, 2.93)	96.1 (< 0.05)	
Adjusted for sex								
Yes	6	1.71 (1.21, 2.42)	79.7% (< 0.05)	**> **0.05	1	1.42 (1.03, 1.95)	NA	**< 0.05**
No	3	1.44 (0.97, 2.15)	65% (> 0.05)		5	1.97 (1.32, 2.93)	96.1 (< 0.05)	
Adjusted for asthma								
Yes	4	1.78 (0.97, 3.24)	85.7% (< 0.05)	**> **0.05	1	1.42 (1.03, 1.95)	NA	**< 0.05**
No	5	1.41 (1.14, 1.75)	71.3% (< 0.05)		5	1.97 (1.32, 2.93)	96.1 (< 0.05)	
Adjusted for atopic dermatitis								
Yes	3	1.86 (1.41, 2.46)	2.6% (> 0.05)	**< 0.05**	0	NA	NA	NA
No	6	1.38 (1.09, 1.74)	78.6% (< 0.05)		6	1.86 (1.32, 2.62)	95.1 (< 0.05)	
Adjusted for household income								
Yes	3	1.69 (1.15, 2.49	68.5% (< 0.05)	**> **0.05	0	NA	NA	NA
No	6	1.55 (1.12, 2.13)	78% (< 0.05)		6	1.86 (1.32, 2.62)	95.1 (< 0.05)	
Adjusted for comorbidities								
Yes	3	1.31 (0.83, 2.05)	78% (< 0.05)	> 0.05	1	1.42 (1.03, 1.95)	NA	**< 0.05**
No	6	1.73 (1.31, 2.3)	82.4% (< 0.05)		5	1.97 (1.32, 2.93)	96.1 (< 0.05)	

A P-value <0.05 was considered significant

The diagnosis modality included in the primary studies also seemed to be related to the results. For example, studies ascertaining AR and depression by diagnosis demonstrated a stronger association between AR and depression incidence (OR: 2.20, 95% CI 1.56–3.08 and OR: 2.19, 95% CI 1.42–3.38, respectively) than the studies using self-reporting (OR: 1.29, 95% CI 1.05–1.57 and OR: 1.34, 95% CI 1.09–1.65, respectively).

The association between AR and depression was strong when the studies were not adjusted for age or adjusted for the presence of atopic dermatitis (Table 2).

#### Rhinitis and the risk of depression

Among the fourteen studies included in our analysis, six articles reported an association between rhinitis and depression. Stratified analyses across several key study characteristics and clinical factors were performed to evaluate the possible sources of heterogeneity in the included studies (Table 2). The association between rhinitis and depression was significant (OR: 1.86, 95% CI 1.32–2.62), and this association was consistent in all stratified analyses (Table 2). The study location, study quality, and sample size seemed to be related to the results. For example, stronger associations between rhinitis and depression were found in studies conducted in Europe (OR: 2.61, 95% CI 1.07–6.37) compared to studies conducted in the United States (OR: 1.85, 95% CI 1.15–2.96) or Asia (OR: 1.42, 95% CI 0.96–2.10), in studies with a small sample size (< 10,000) (OR: 2.10, 95% CI 1.51–2.90) compared to studies with a large sample size (≥ 10,000) (OR: 1.42, 95% CI 0.96–2.10), and in studies with a NOS score ≤ 5 (OR: 2.37, 95% CI 1.66–3.39) compared to those with a NOS score > 5 (OR: 1.39, 95% CI 1.09–1.78) (Table 2).

The association between rhinitis and depression was stronger in the studies unadjusted for age, asthma, and comorbidities or adjusted for fewer confounding factors (< 8) (Table 2).

### Publication bias

**Fig. 3:**
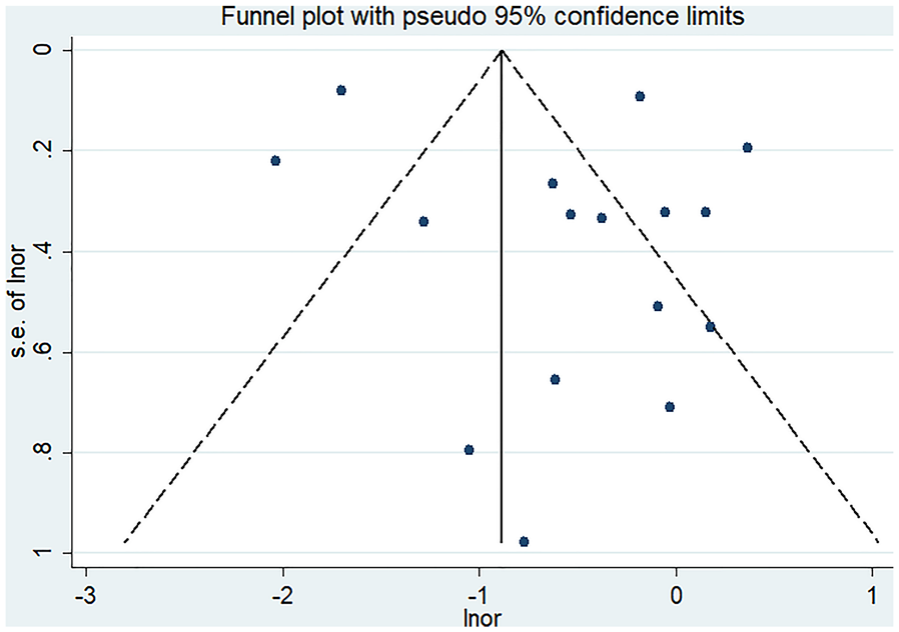
Funnel plot of publication bias in the association between any type of rhinitis and depression

Potential publication bias was revealed by asymmetry funnel plots (Fig. 3). However, Begg’s test was not statistically significant (z = 0.95, *p* = 0.344) (10.17632/ccvm3cvbtm.2).

## Discussion

To the best of our knowledge, this study presents the first meta-analysis to evaluate the association between rhinitis, including AR and NAR, and the incidence of depression. The results of this meta-analysis, which included 14 studies, revealed that any type of rhinitis was associated with a 67% increased risk of depression. Furthermore, our analysis revealed that AR was associated with a 54% increased risk of depression and that NAR was associated with a 115% increased risk of depression. The association persisted and remained statistically significant in all stratified analyses.

The results of this meta-analysis reveal that the patients with NAR were more likely to experience depression than the patients with AR. There are several reasons for this result. First, only two studies [7, 35] with relatively small numbers of participants evaluated the association between NAR and depression; therefore, the association may be overestimated. The sample size may affect the results of the meta-analysis in the stratified analyses (Table 2). Second, depression occurs at an increased frequency in individuals with diseases with prolonged progression, such as chronic respiratory diseases, asthma, chronic atopic dermatitis, cardiovascular diseases and other systemic comorbidities [40, 41]. The patients with NAR had an increased likelihood of a generally poor health status, and these confounding factors were not adjusted in the study by Bedolla-Barajas et al. [35]. Third, the patients with NAR had an increased likelihood of developing nasal obstruction and rhinorrhoea, and these specific presenting symptoms may lead to depression in NAR patients [7]. Fourth, the chronicity of NAR may increase the rate of depression. For example, patients with AR may present with seasonal symptoms, while patients with NAR constantly experience symptoms [7]. Collectively, future prospective cohort studies with large sample sizes are needed to clarify the results by considering additional confounding factors.

The association among AR, rhinitis and depression was significant (Table 2). Patients with AR are prone to allergic disorders with a predominance for the differentiation of CD4-positive T helper 2 (Th2) cells. AR with neuroinflammatory markers may trigger allergies caused by IL-4, IL-5 and IL-6 and, thus, affect psychopathology [5, 42, 43], such as depression, that may be associated with increased inflammatory markers [44]. The association between AR and depression was stronger in the participants in Europe [5] and other countries [35] than in those in Asia [1, 6, 14, 36] and the United States [7, 13, 17] in the stratified analysis (Table 2). Genetic and environmental factors may affect mood [5]. The study location may have affected the results via other confounding factors, such as genes, subjective health status, residential area, and occurrence of allergic disease. The association among AR, rhinitis and depression was slightly stronger in the studies with small sample sizes (˂10,000) than in those with large sample sizes (≥ 10,000). Thus, studies with small numbers of participants may have overestimated the association among AR, rhinitis and depression.

The different methods of ascertaining depression and AR seemed to be related to the results. The studies in which depression and AR were diagnosed by physicians reported a slightly stronger association than those in which depression and AR were self-reported, and the studies with self-reporting may have been subject to recall bias [5].

Furthermore, many confounding factors are related to the results. Patients with AR often also suffer from asthma and atopic dermatitis [5, 19]. Age, asthma, atopic dermatitis, and sex were found to be related to an increased risk of depression in the stratified analysis. The studies considering fewer than 8 confounding factors may have overestimated the association between AR and depression. Future studies are needed to clarify these associations by considering additional confounding factors.

Our meta-analysis has the following limitations. First, the studies included a wide range of participants, including children and adults, in different age groups, which could result in biases. Second, some included studies reported the association between any type of rhinitis and depression without adjusting for confounding factors, such as the crude ORs or number of participants, which may have led to an overestimation of the results of the meta-analysis. Third, the treatment options for rhinitis, AR and NAR may impact their risk of expressing depressive symptoms, but the original included studies did not discuss this issue. Fourth, potential publication bias exists because we included only studies published in English. Fifth, studies reporting outcomes in alternative manners were not included in our meta-analysis, which may contribute to publication bias. For example, studies that did not evaluate the association between rhinitis and depression by ORs or conversion of ORs were excluded. Sixth, there was no analysis of the association between different types of rhinitis and different degrees of depression based on the original studies. Furthermore, bias inherent to observational studies was not eliminated in the quantitative synthesis.

The merits of this meta-analysis are as follows. First, this study evaluated the association between rhinitis and depression in a large global sample. Considering the consistent finding of an increased depression incidence associated with rhinitis, we recommend that further prospective cohort studies considering additional adjusted confounding factors should be performed to test this hypothesis. Second, this study demonstrated that the study location, study design, sample size, ascertainment of depression and rhinitis, and adjustment for confounding factors were all sources of heterogeneity.

## Conclusions

In conclusion, our pooled analyses provide evidence that participants with rhinitis, including AR and NAR, had an increased risk of depression. Future studies may focus on treatment options for rhinitis and explore how AR and NAR impact the risk of expressing depressive symptoms.

## Supplementary Information

**Additional file 1.** MOOSE checklist for meta-analyses of observational studies.

**Additional file 2.** Newcastle–Ottawa Quality Assessment Scale results for cohort and cross-sectional studies.

**Additional file 3.** Cumulative evidence for association of rhinitis and depression.

## Data Availability

No additional data are available.

## Abbreviations

AR: Allergic rhinitis
BASC-2: Behavior Assessment System for Children, Second Edition
BDI-II: Beck Depression Inventory-II
CCAAPS: Cincinnati Childhood Allergy and Air Pollution Study
CI: Confidence interval
EPOS: European Position Paper on Rhinosinusitis and Nasal Polyps
HADS: Hospital Anxiety and Depression score
ICD-9-CM: International Classification of Disease, 9th Revision, Clinical Modification
K-6: Kessler’s K-6 Non-Specific Psychological Distress Scale
KNHIS-NSC: National Sample Cohort of the Korea National Health Insurance Service
TAD: Testing for depression and anxiety characteristics using the Depression and Anxiety in Youth Scale
KNHANES: Korean National Health and Nutrition Examination Survey
NA: Not applicable because only 1 study
NAR: Nonallergic rhinitis
NFO: National Family Opinion
NHANES: National Health and Nutrition Examination Survey
NHIRD: National Health Insurance Research Database
NHIS: National Health Interview Survey
NHIRD: National Health Insurance Research Database
NPHS: National Population Health Survey
OR: Odds ratio
QOL: Quality of life
TEMPS-A: Temperament Evaluation of Memphis, Pisa, Paris, France, San Diego Auto-questionnaire
