# Supplementary material for: Cumulative evidence for association of rhinitis and depression
Source: Allergy Asthma Clin Immunol. 2021 Oct 24;17:111. doi: 10.1186/s13223-021-00615-5 (PMC8543924; doi:10.1186/s13223-021-00615-5)
Supplement: Supplementary file 2 — Additional file 2. Newcastle–Ottawa Quality Assessment Scale results for cohort and cross-sectional studies. [file 13223_2021_615_MOESM2_ESM.docx]

**Newcastle - Ottawa Quality Assessment Scale results for cohort and cross-sectional studies**

|  | **Selection** | | | | **Comparability** | **Outcome** | | |  |
| --- | --- | --- | --- | --- | --- | --- | --- | --- | --- |
| **Study** | **Representativeness of the Exposed Cohort** | **Selection of the Non-Exposed Cohort** | **Ascertainment of Exposure** | **Demonstration That Outcome of Interest Was Not Present at Start of Study** | **Comparability of Cohorts on the Basis of the Design or Analysis** | **Assessment of Outcome** | **Was Follow-Up Long Enough for Outcomes to Occur** | **Adequacy of Follow Up of Cohorts** | **Score** |
| Zhou 2017 | ****** | ***** | ***** | **/** | ***** | ***** | **/** | **/** | **6** |
| Yamamoto-Hanada, K. 2019 | ****** | ***** | ***** | **/** | ***** | ***** | **/** | **/** | **6** |
| Roxbury, C. R 2019 | ****** | ***** | ***** | **/** | ***** | ***** | **/** | **/** | **6** |
| Kim, D. H. 2016 | ****** | ***** | ***** | **/** | ***** | ***** | **/** | **/** | **6** |
| Nanda, M. K. 2016 | ***** | ***** | ***** | ***** | ***** | ***** | ***** | ***** | **8** |
| Chen, M. H.2013 | ***** | ***** | ***** | ***** | ***** | ***** | ***** | **/** | **7** |
| Bedolla-Barajas, M 2017 | ***** | ***** | ***** | **/** | ***** | ***** | **/** | **/** | **5** |
| Valero, A. 2015 | ***** | ***** | ***** | **/** | ***** | ***** | **/** | **/** | **5** |
| Wei, H. T. 2016 | ***** | ***** | ***** | **/** | ***** | ***** | ***** | ***** | **7** |
| Audino, P. 2014 | ***** | ***** | ***** | **/** | ***** | ***** | **/** | **/** | **5** |
| Derebery, J.2008 | ****** | ***** | ***** | **/** | ***** | ***** | **/** | **/** | **6** |
| Tas, H. I. 2019 | ***** | ***** | ***** | **/** | ***** | ***** | **/** | **/** | **5** |
| Shin, J. H.2018 | ****** | ***** | ***** | **/** | ***** | ***** | **/** | **/** | **6** |
| Seo, J.2012 | ***** | ***** | ***** | **/** | ***** | ***** | **/** | **/** | **5** |
