# Supplementary material for: Cumulative evidence for association of rhinitis and depression
Source: Allergy Asthma Clin Immunol. 2021 Oct 24;17:111. doi: 10.1186/s13223-021-00615-5 (PMC8543924; doi:10.1186/s13223-021-00615-5)

**Cumulative evidence for association of rhinitis and depression**

**Text S1. Retrieval strategy of PubMed, EMBASE and Cochrane Library databases**

Before 1 April 2019.

**PubMed 331**

Search (((((((NARES[Title/Abstract]) OR NAR[Title/Abstract]) OR LAR[Title/Abstract]) OR NANIPER[Title/Abstract])) OR ((rhinitis[Title/Abstract]) OR rhinit*[Title/Abstract]))) AND ((((depress*[Title/Abstract]) OR melancholia[Title/Abstract])) OR ((((Depression[Title/Abstract]) OR depressive disorder[Title/Abstract]) OR depressive disorder, major[Title/Abstract]) OR dysthymic disorder[Title/Abstract]))

**EMBASE 2995**

1. exp rhinitis/

2. "rhinit*".tw.

3. (NARES or NAR or LAR or NANIPER).tw.

4. 1 or 2 or 3

5. Depression/

6. depressive disorder/ or depressive disorder, major/ or dysthymic disorder/

7. (depress* or melancholia).ab,ti.

8. 5 or 6 or 7

9. 4 and 8

**Cochrane Central Register of Controlled Trials (CENTRAL, The Cochrane Library) 146**

#1 MeSH descriptor Depression, this term only

#2 MeSH descriptor Depressive Disorder, this term only

#3 MeSH descriptor Depressive Disorder, Major, this term only

#4 MeSH descriptor Dysthymic Disorder, this term only

#5 (depress* or melancholia)

#6 (#1 OR #2 OR #3 OR #4 OR #5)

#7 MeSH descriptor: [Rhinitis] explode all trees

#8 rhinit*

#9 NARES or NAR or LAR or NANIPER

#10 (#7 OR #8 OR #9)

#11 (#6 AND #10)

**Text S2. Egger's and Begg’s test for publication bias test**

Tests for Publication Bias

Begg's Test

adj. Kendall's Score (P-Q) = -22

Std. Dev. of Score = 22.21

Number of Studies = 16

z = -0.99

Pr > |z| = 0.322

z = 0.95 (continuity corrected)

Pr > |z| = 0.344 (continuity corrected)

Egger's test

------------------------------------------------------------------------------

Std_Eff | Coef. Std. Err. t P>|t| [95% Conf. Interval]

-------------+----------------------------------------------------------------

slope | -1.168228 .3248418 -3.60 0.003 -1.864944 -.4715112

bias | 1.821312 1.609735 1.13 0.277 -1.631226 5.273851

------------------------------------------------------------------------------

**Text S3 Begg’s and Egger's test for publication bias test**


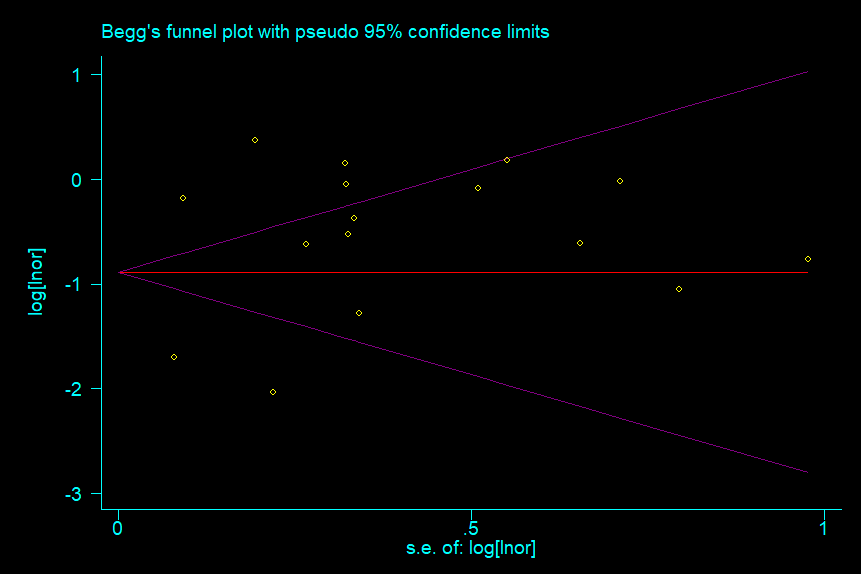

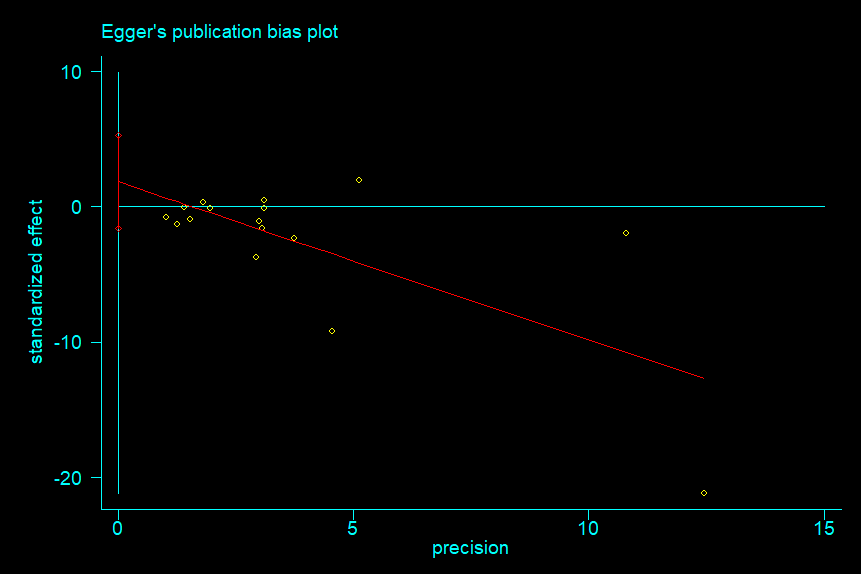

Supplement: Supplementary file 3 — Additional file 3. Cumulative evidence for association of rhinitis and depression. [file 13223_2021_615_MOESM3_ESM.doc]
